# Micromanagement of Lymphomas

**DOI:** 10.1371/journal.pbio.0060156

**Published:** 2008-06-24

**Authors:** Enrico Arpaia, Tak W Mak

## Abstract

B cell receptor signaling participates in the genesis of lymphoma and influences the characteristics of the tumor cells.

Lymphomas are tumours composed of lymphocytes. The two types of lymphocytes—T cells and B cells—are distinguished by the antigen receptors on their surfaces and the specific functions they undertake on behalf on the immune system. Precursors of B cells are generated in the bone marrow and leave only after they have undergone a genetic rearrangement of the DNA encoding the protein chains that compose the functional B cell receptor (BCR). Each BCR binds a specific antigen, and if it is a foreign antigen (i.e., from an invading pathogen), that specific B cell undergoes division to produce many identical progeny (clonal amplification) that will later produce antibodies, which are the secreted form of the BCR present on the cell surface.

Normal pre-B lymphocytes are developmentally programmed to undergo apoptosis (Bcl-2 gene expression, which results in survival, is down-regulated in these cells). However, the expression of a functional BCR leads to signalling that up-regulates Bcl-2 expression and rescues these cells such that they relocate in the peripheral blood and become mature B cells [1–4]. Therefore, during development, B cells are continuously under selective pressure to express functional BCRs. This implies the existence of a basic BCR-mediated signal that provides maintenance of the B cell homeostasis [5]. The nature of this constitutive signal is distinct from an antigen-driven signal that leads to proliferation and clonal expansion of the mature B cells, and therefore it is better defined as a basal, or “tonic”, signal [6–8]. However, a mechanistic understanding of this survival tonic signal is still lacking. Perhaps B cells require constitutive low-level receptor engagement with low-affinity autoantigens for survival [9]. Conversely, the tonic signal could be the result of a steady-state level of signalling in unstimulated cells, generated by an equilibrium between positive and negative regulators downstream of the BCR [8]. Certainly, the specific signalling pathway initiated by the BCR to sustain pre-B cells is still elusive, and it remains debatable whether the receptor signals autonomously or requires activation by antigen.

## Role for the BCR in Lymphoma Induction—The Evidence

One fascinating aspect of BCR signalling is its potential involvement in lymphomagenesis. Many B cell lymphomas are caused by reciprocal chromosomal translocations that result in an oncogene coming under the control of an active antibody (immunoglobulin, Ig) gene locus. Deregulated oncogene expression then leads to constitutive transcription/translation and eventually transformation of the cell to a cancerous state. The fact that most B cell lymphoma cells express a functional BCR raises several interesting questions. Is the BCR required for lymphomagenesis? Does the BCR contribute to tumour cell proliferation? Does a tonic signal or an encounter with its matching (cognate) antigen augment BCR signalling leading to lymphomagenesis?

In a new PLoS Biology study, Refaeli et al. ask precisely these questions and present evidence (summarized in Table 1) that the BCR plays a pivotal role in lymphomagenesis [10]. To reach their conclusions, Refaeli et al. took advantage of EμMYC mice, which bear a transgene expressing the MYC oncogene under the control of the enhancer (μ) in the IgH locus. EμMYC mice have long been known to develop clonal tumours of pre-B or B cells [11,12]. Refaeli et al. generated a series of derivative EμMYC transgenic mice. Table 2 summarizes the characteristics of these mice. When Refaeli et al. characterized the tumours developing in these mutant strains, they found that EμMYC/sHEL mice developed lymphomas at the same rate as EμMYC mice. Thus, the continuous presence of a specific antigen (hen egg lysozyme (HEL)) alone does not alter the cancer phenotype of the mice. Intriguingly, the introduction of BCR^HEL^ alone accelerated the onset of lymphomas compared to the rate of onset in EμMYC and EμMYC/sHEL mice. The introduction of BCR^HEL^ concomitant with continuous production of the HEL antigen produced a further acceleration of lymphomagenesis compared to EμMYC/BCR^HEL^ mice. These data clearly demonstrate that the BCR can cooperate with the MYC oncogene to accelerate lymphomagenesis, and that this acceleration is increased when the BCR is stimulated by cognate antigen. Thus, a possible interpretation of the data is that the presence alone of a specific BCR (BCR^HEL^) seems to intensify the effect of the tonic signal and, when the specific BCR^HEL^ and its cognate sHEL antigen are present, the tonic signal becomes a full strength signal.

**Table 1:**
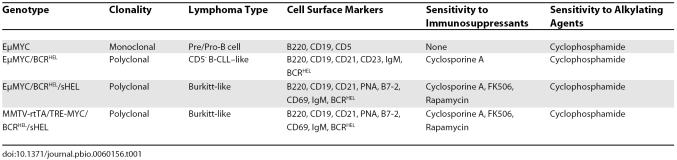
Phenotypes of EμMYC derivative strains created by Refaeli et al.

**Table 2:**
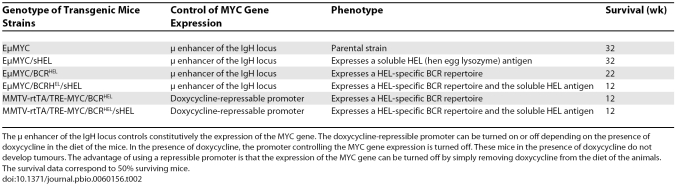
EμMYC-Derived Transgenic Strains

**Figure 1:**
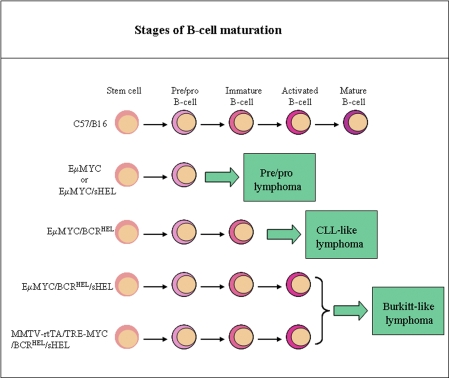
Cellular Derivation of the Lymphomas in EμMYC and EμMYC-Derived Mouse Strains In Refaeli et al.'s experimental system [10], mice of different genotypes develop lymphomas at different stages of B cell maturation in response to different antigenic stimuli. EμMYC mice develop oligoclonal pre/pro-B cell lymphomas in response to nonspecific antigens, generating a tonic signal that promotes survival (see text). EμMYC/BCRHEL mice express a transgenic BCR that mediates an enhanced tonic signal; these mice develop more mature polyclonal CLL-like lymphomas. B cells of EμMYC/BCR^HEL^/sHEL and MMTV-rtTA/TRE-MYC/BCR^HEL^/sHEL mice receive the most powerful “full-strength signal” when they encounter specific HEL antigen; these mutants develop polyclonal, more mature B cell lymphomas. In all these cases, tumorigenesis is dependent on the overexpression of *MYC* and on continuous BCR stimulation. The phenotype of the tumours developed correlates with the strength of the antigenic stimulus.

Curiously, different derivative EμMYC strains developed different types of tumours. Figure 1 depicts the cellular derivation of these tumours. Lymphomas of EμMYC mice are characteristically pre/pro-B cell in nature, but the tumours in EμMYC/BCR^HEL^ mice contained more mature but naive CD5^−^ cells. In chronic lymphocytic leukemia (CLL) approximately 95% of the cells express a B-phenotype (B-CLL) and express the CD5 antigen. However, among B CLL, 7%–20% are CD5^−^ [13]. The significance of the absence of CD5 expression in these cells is unclear; however, CLL with low expression of CD5 should be regarded as a subtype of CLL [13]. Therefore, the tumours in EμMYC/BCR^HEL^ should be classified as CLL. In contrast, EμMYC/BCR^HEL^/sHEL mice developed tumours similar to Burkitt lymphomas. Another surprising difference was that, although lymphomas of EμMYC mice were largely monoclonal in nature, the tumours isolated from EμMYC/BCR^HEL^, EμMYC/BCR^HEL^/sHEL, and MMTV-rtTA/TRE-MYC/BCRHEL/sHEL mice were polyclonal. For example, EμMYC/BCR^HEL^ and EμMYC/BCR^HEL^/sHEL exhibited 20–40 clones and 10–15 clones, respectively. This is somewhat surprising because EμMYC tumours are monoclonal, and human B lymphomas are generally monoclonal [14,15]. In fact, clonality can be clinically used to distinguish between a chronic inflammatory hyperproliferation and a neoplasm [15].

How the introduction of a specific BCR repertoire in the EμMYC background leads to multiclonality is not clear. The question arises as to whether the lymphoma is a true tumour or if it is the result of excessive proliferation. However, tumours from all of the derivative EμMYC mutants were transplantable into recipient mice. This demonstrates that indeed the donor cells were tumour cells. These transplantation experiments showed that antigen stimulation of the BCR is required for B cell transformation. Mice transgenic for MMTV-rtTA/TRE-MYC/BCR^HEL^ did not develop tumours if they were treated with doxycycline to repress MYC expression. When the sHEL-recognizing B cells from these mice were transplanted into C57/B6 recipients, tumours did not develop even in the absence of doxycycline (when the MYC gene is expressed constitutively) due to the lack of sHEL expression. However, when these same cells were transplanted into HEL-expressing mice, tumours readily appeared. Furthermore, these tumours were transplantable in HEL-expressing animals but not in wild-type animals, even in the absence of doxycycline. Refaeli et al. also noted that tumour cells from either EμMYC/BCR^HEL^/sHEL or MMTV-rtTA/TRE-MYC/BCR^HEL^/sHEL mice generated lethal tumours in recipients that did not express the HEL antigen, but this was attributed to the expression of the HEL transgene by the transplanted tumour cells. These in vivo experiments clearly support the notion that both expression alone of the BCR, as well as BCR binding to its cognate antigen, are required to promote lymphomagenesis. Interestingly, these two mechanisms appear to operate independently, because EμMYC/BCR^HEL^ and EμMYC/BCRHEL/sHEL mice develop different types of tumours.

## A Block in BCR Signalling Blocks Lymphoma Generation

Another experiment supporting the requirement for BCR signalling in lymphomagenesis involved the silencing of signalling components of the BCR—Iga/Igß. Tumours isolated from EμMYC/BCR^HEL^ mice were transduced with lentivirus encoding shRNA directed against either Iga or Igß. The transduced tumour cells were then transplanted into Rag^−/−^ mice that were incapable of any T cell responses to the virus (and thus also incapable of any T-dependent B cell responses). In the absence of Iga/Igß signalling, the transplanted tumours failed to expand in the immunodeficient recipients. This result not only confirms the role of BCR signalling in lymphomagenesis but also implies that continuous signalling by the BCR is required for the tumour to thrive.

If BCR signalling is truly crucial for the formation of tumours in EμMYC/BCR^HEL^, EμMYC/BCR^HEL^/sHEL, and MMTV-rtTA/TRE-MYC/BCR^HEL^/sHEL mice, then these lymphomas should be sensitive to immunosuppressive drugs that block the BCR signalling pathway at particular points. Refaeli et al. transplanted recipient mice with tumours from the derivative EμMYC strains and treated these recipients with the immunosuppressants cyclosporine A, FK506, and rapamycin. They then compared tumour growth in these animals with growth in tumour-transplanted recipients treated with the more general inhibitor cyclophosphamide. Cyclosporine A is thought to bind to the cytosolic protein cyclophilin expressed by all immunocompetent lymphocytes. This cyclosporine A/cyclophilin complex inhibits calcineurin, which normally activates interleukin-2 (IL-2) transcription and sustains effector T cell functions. FK506 reduces peptidylprolyl isomerase activity by binding to the immunophilin FKBP-12, creating a new complex. This FKBP-12/FK506 complex also interacts with and inhibits calcineurin, again blocking IL-2 transcription. Rapamycin binds to cytosolic FKBP-12 in a manner similar to FK506 but forms a rapamycin/FKBP-12 complex that binds directly to mTOR complex 1, disrupting the mammalian target of rapamycin (mTOR) pathway. Lymphocyte responses to IL-2 are thus decreased, and T and B cell activation is abrogated. In contrast to these signalling inhibitors, cyclophosphamide acts mainly on a cell's DNA via the metabolite phosphoramide mustard, which forms lethal DNA crosslinks at guanine N-7 positions. In Refaeli et al.'s experiments, the growth of tumours in EμMYC mice was inhibited only by cyclophosphamide. However, tumours in EμMYC/BCR^HEL^ mice responded to either cyclosporine A or cyclophosphamide. Tumours in EμMYC/BCR^HEL^/s^HEL^ and MMTV-rtTA/TRE-MYC/BCR^HEL^/sHEL mice responded to all immunosuppressive drugs tested as well as to cyclophosphamide. It is not clear why EμMYC/BCR^HEL^ and EμMYC/BCR^HEL^/sHEL or MMTV-rtTA/TRE-MYC/BCR^HEL^/sHEL tumours respond differently to immunosuppressants. However, BCR signalling definitely drives some lymphomas, and the signalling emanating from an unoccupied BCR may be different from that triggered by a BCR engaged by cognate antigen.

## The Tonic/Full-Strength BCR Signalling Hypothesis

It is not hard to imagine that BCR functions would be similar during normal B cell development and lymphomagenesis. Current theory holds that, in normal animals, the positive selection of mature B cells depends on BCR signalling. In addition, the BCR provides a weak but essential survival stimulus to a mature B cell in the periphery, while this cell is awaiting an encounter with cognate antigen. Non-cognate antigens that briefly and nonspecifically “tickle” the BCR may initiate a tonic signal that mediates survival.

However, it is not until BCR engagement by cognate antigen that the receptor delivers a full-strength signal to the B cell that leads to activation of the transcription factor NF- B followed by proliferation and differentiation. A parallel series of events may occur during lymphomagenesis, as is illustrated by Refaeli et al.'s EμMYC-derivative mice.

The exact identity of this tonic signal remains obscure. It may involve a trickle of survival signals from the phosphatidylinositol 3-kinase (PI3K), NF- B, or BCL-2 pathways. Here, we postulate its existence and define it operationally. Basal activation of the diverse BCR repertoire of EμMYC mice may provide a tonic signal that can confer survival to the first transformed B cell. A similar kind of signal has been postulated to overcome the intrinsic homeostatic cell death mechanism mediated by cytochrome C release (a protein that is released by the mitochondria in response to pro-apoptotic signals) or death receptor engagement [16]. In the case of EμMYC/BCR^HEL^ mice, the transgenic BCRHEL BCR appears to deliver an enhanced tonic signal. This signal attains full-strength in EμMYC/BCR^HEL^/sHEL mice when the BCR is engaged by cognate antigen. In all three types of mutants, when *MYC* expression becomes deregulated such that *MYC* is over expressed, the transformed B cell proliferates and starts to form a tumour (Figure 1). One puzzle that remains is why do EμMYC/BCR^HEL^ mice exhibit accelerated lymphomagenesis in the absence of HEL antigen? It may be that the putative enhanced tonic signal delivered by the transgenic BCR^HEL^ leads to prolonged or stronger NF- B signalling, which in turn accelerates tumour cell proliferation. NF- B can be activated in a myriad of signal- and cell-specific ways [17], and different pathways affect the intensity of the signal delivered.

A human example that is partially consistent with the tonic/full-strength BCR signalling hypothesis may be the development of MALT lymphomas, which are B cell malignanices in the mucosa-associated lymphoid tissues. Gastric lymphomas are thought to be initiated by Helicobacter pylori infections that stimulate the hyperproliferation of B cells specific for H. pylori antigens. The chronic inflammation induced by persistent H. pylori infection may cause DNA damage leading to genetic abnormalities and the emergence of a neoplastic B clone. Three such genetic abnormalities are recurrent chromosomal translocations. T(11;18)(q21;q21) results in the expression of an API2-MALT1 fusion protein [18]. The API2 gene product is an apoptosis inhibitor, which inhibits the activity of caspase 3, 7, and 9. T(1;14)(p22;q32) and T(14;18)(q32;q21) cause the BCL10 and MALT1 genes, respectively, to come under the control of the IgH locus, dysregulating their expression [18]. The BCL10 and MALT1 proteins are components of the antigen receptor signalling pathway that leads to NF- B activation [17]. In early-stage gastric MALT lymphomas, tumour growth is stimulated by H. pylori antigens and direct CD40-mediated interaction between T and B cells [19]. However, when the translocated gene sequences are expressed, lymphoma growth becomes independent of H. pylori and BCR stimulation. The analogy between Refaeli et al.'s experimental system and MALT lymphomas lies in the fact that the BCR delivers survival signals to B cells that give rise to tumours.

## Future Directions

The BCR activates several signal pathways, including the phosphatidylinositol 3-kinase (PI3K) pathway [20]. However, the similarities between Refaeli et al.'s experimental system and MALT lymphomas suggest that BCL10 and NF- B may be involved in the lymphomagenesis occurring in EμMYC/BCR^HEL^/sHEL and MMTV-rtTA/TRE-MYC/BCR^HEL^/sHEL mice. Therefore, further investigation of this pathway may yield novel information on the relationship between immune responses and lymphomas. In addition, knowledge of the precise signalling pathway driving a particular lymphoma has the potential to improve treatment by allowing highly specific and effective therapies to be deployed.

## Abbreviations

BCR: B cell receptor
CLL: chronic lymphocytic leukemia
HEL: hen egg lysozyme
Ig: immunoglobulin
IL-2: interleukin 2
